# The Mediating Effect of Concurrent Changes in Dietary Behaviors on the Associations Between Intervention and Changes in Adiposity Outcomes: Evidence from a Cluster-Randomized Controlled Trial

**DOI:** 10.3390/nu17030376

**Published:** 2025-01-21

**Authors:** Hai-Xue Wang, Lan Cheng, Xin Yuan, Jin-Lang Lyu, Ping Li, Shi-Yu Yan, Hui Wang, Yan-Sheng Ding, Shen-Da Hong, Hai-Jun Wang

**Affiliations:** 1 National Institute of Health Data Science at Peking University, Peking University, Beijing 100191, China; gwwanghaixue@126.com; 2 Institute of Medical Technology, Health Science Center of Peking University, Beijing 100191, China; 3 Department of Maternal and Child Health, School of Public Health, Peking University, National Health Commission Key Laboratory of Reproductive Health, Beijing 100191, China; uaninn@163.com (X.Y.); jinlanglyu@bjmu.edu.cn (J.-L.L.); 1810306241@pku.edu.cn (P.L.); yan_sy@bjmu.edu.cn (S.-Y.Y.); huiwang@bjmu.edu.cn (H.W.); 4 MRC Centre for Environment and Health, Department of Epidemiology and Biostatistics, School of Public Health, Imperial College London, London W12 0BZ, UK; lan.cheng@imperial.ac.uk; 5 Weifang Maternal and Child Health Hospital, Weifang 261000, China; ydukongjian@163.com; 6 Peking University Health Science Center, Weifang Joint Research Center for Maternal and Child Health, Beijing 100191, China

**Keywords:** children, obesity, intervention, dietary behavior, change, mediating effect

## Abstract

Behavioral interventions have been shown to be effective in improving dietary behavior and reducing childhood obesity. There is limited evidence on how concurrent changes in dietary behavior from intervention studies affect childhood obesity. The present study aimed to evaluate the mediating effect of concurrent changes in dietary behaviors between the intervention and changes in adiposity indicators. This study included 1180 children from the DECIDE-Children study, which was conducted across three areas in China, aiming to promote children’s healthy diet and physical activity, while also engaging schools and families to support children’s behavioral changes. Dietary behaviors were collected by a revised version of the Food Frequency Questionnaire and a self-designed questionnaire. Adiposity outcomes were objectively measured by trained personnel. Generalized linear mixed models were used to estimate the association between scores of dietary behavioral changes and adiposity indicator changes. Mediation analyses were used to evaluate how scores of dietary behavioral changes mediated the effect of intervention on adiposity indicator changes. Six hundred children in the intervention group and five hundred and eight in the control group with both baseline and follow-up data were included. Each increase in dietary behavioral change score was associated with a 0.06 (*p* = 0.016) decrease in changes in BMI and other adiposity indicators. Scores of dietary behavioral changes mediated 13.87% (*p* < 0.001), 11.81% (*p* < 0.001), 17.60% (*p* = 0.024), and 16.78% (*p* = 0.032) of the association between intervention and changes in body mass index (BMI), BMI z-score, body fat percentage, and waist circumference, respectively. Scores of dietary behavioral changes mediated the intervention effect on adiposity indicator changes. Future interventions targeting childhood obesity should incorporate promoting multiple dietary behaviors simultaneously.

## 1. Introduction

Childhood overweight and obesity has been a global public health concern. In 2020, 430 million children and adolescents aged 5 to 19 years were affected by overweight and obesity, representing 22.0% of the global child population, which is expected to rise to 39.0% by 2035 [1]. China has been experiencing an increasing trend in overweight and obesity among children aged 5–19 years, with the prevalence having arrived at 37.0% in 2020 [1]. Childhood obesity is a risk factor for several adverse health outcomes, including type 2 diabetes mellitus, hypertension, fatty liver disease, and psychosocial complications [2], which leads to increased economic costs for individuals and society [3]. Therefore, feasible and effective interventions are urgently needed to prevent childhood obesity.

In the past few decades, the exponential global expansion of fast-food restaurants and the saturation of large chain supermarkets have enhanced the accessibility of unhealthy foods characterized by high-calorie content, excessive processing, substantial portion sizes, sugary beverages, and unhealthy fats [4]. A large amount of evidence has indicated an increase in the consumption of sugar-sweetened soft drinks, junk foods, fast food, and carbohydrates among children and adolescents [5,6,7,8]. Evidence from prospective cohort studies and randomized controlled trials (RCTs) has shown that increases in unhealthy dietary behaviors, including consumption of sugar-sweetened beverages, unhealthy snacks, Western fast food, and eating out are associated with childhood obesity [9,10,11]. The compelling evidence has prompted numerous childhood obesity intervention studies to emphasize dietary behavior change as a key component, and has consistently shown its significant effect on preventing overweight and obesity in children [12,13,14,15,16,17,18].

However, the majority of intervention studies have only focused on evaluating the intervention effect on modifying unhealthy dietary behaviors and obesity-related outcomes separately, with no evidence analyzing the association between concurrent changes in multiple dietary behaviors and adiposity outcomes among children. There is only some evidence that attempts to explore the mechanisms by which dietary factors influence the effectiveness of interventions from other aspects. A previous multidisciplinary childhood obesity treatment study assessed whether the treatment effect in childhood obesity was associated with the consumption of sweetened beverages, candy, unhealthy snacks, or fast food at baseline, indicating that children’s baseline dietary behaviors were not associated with the degree of weight loss during treatment [19]; evidence that excessive intake of saturated fat was associated with obesity may contribute to explaining the results [20]. However, the study did not analyze the relationship between concurrent behavioral changes and weight loss. Another previous study examined the association between single lifestyle behavior changes and weight outcomes among preschoolers receiving intervention for obesity, suggesting that only reductions in absolute caloric intake were significantly associated with reductions in BMI z-score. However, it also did not explore the relationship between concurrent changes in lifestyle behaviors at the end of the trial and weight outcomes [21]. In addition, the method of calculating a composite score to assess the combined impact of various factors on health outcomes has been widely applied [22]. For example, dietary patterns demonstrated by scores of different foods, nutrients, and beverages have been widely used to represent the overall effect of diet on the risk of health outcomes, and prospective cohort studies have revealed that higher healthy dietary pattern scores measured by the Planetary Health Diet Index were associated with lower risks of major cardiovascular diseases, type 2 diabetes, cancer, and mortality [23,24]. Inspired by these practices, we believe it is necessary to calculate a composite score to reflect the overall dietary behavioral changes and examine whether children with higher scores of dietary behavioral changes are associated with greater decreases in adiposity indicators.

Furthermore, the mediating role of concurrent changes in dietary behaviors in the associations between intervention and changes in adiposity indicators in children remains unclear. However, evidence from our previous studies has identified that changes in higher-bound scores of the Diet Balance Index Revision [25] and body image [26] mediated the associations between the intervention and changes in BMI and other adiposity indicators in school-age children. Understanding these mechanisms is essential not only for deciphering how interventions exert their effects but also for informing future design of more efficient and targeted obesity prevention strategies for children. The strong plasticity of children’s dietary choice behavior caused by their developing olfactory and gustatory sensitivity [27], as well as the low cost of behavioral intervention [28], make the behavioral interventions to prevent childhood obesity more feasible. Therefore, clarifying the mediating effects of dietary behavior modifications is of great significance.

Our research team has published a rigorously designed RCT of an obesity intervention: the Diet, Exercise and Cardiovascular Health–Children (DECIDE-Children) study [17]. It demonstrated that multicomponent interventions including dietary and physical activity behavior component could effectively reduce both mean body mass index (BMI) and obesity prevalence, as well as improve multiple unhealthy dietary behaviors simultaneously in primary school children, but it did not improve children’s daily moderate-to-vigorous physical activity (MVPA) behaviors. Based on the effectiveness of the obesity intervention observed in our previous DECIDE-Children study, the present study focused on elucidating the relationship between concurrent changes in dietary behavior and changes in obesity indicators, and examining the potential mediating role of concurrent changes in dietary behaviors linking intervention and adiposity indicators. The goal is to provide scientific evidence on promoting changes of dietary behaviors in multifaced interventions for childhood obesity in the future.

## 2. Materials and Methods

### 2.1. Study Design and Participants

The DECIDE-Children study was a multifaceted cluster RCT. This study was conducted across three socioeconomically distinct areas in China (Beijing, Changzhi of Shanxi Province, and Urumuqi of Xinjiang Province), including 24 participating schools with 8 schools from each area. After the baseline survey at September 2018, the schools were randomly allocated at a 1:1 ratio to either the intervention or control group. Randomization was stratified by district within each area and performed by an independent researcher at Peking University who was blinded to the schools. About 50 children aged 8 to 10 years in Grade 4 from each school were recruited. Schools with a number of Grade 4 students greater than 50, with the principal agreeing to the randomization procedure and complying with the study protocol, were eligible for inclusion. Students aged 8 to 10 years old, with parents and/or guardians signing the written informed consent, and meeting health status criteria were eligible for inclusion. More details have been published previously in the protocol and related articles [17,29]. Ethical approval was obtained by the Peking University Institutional Review Board (IRB00001052-18021).

**Figure 1:**
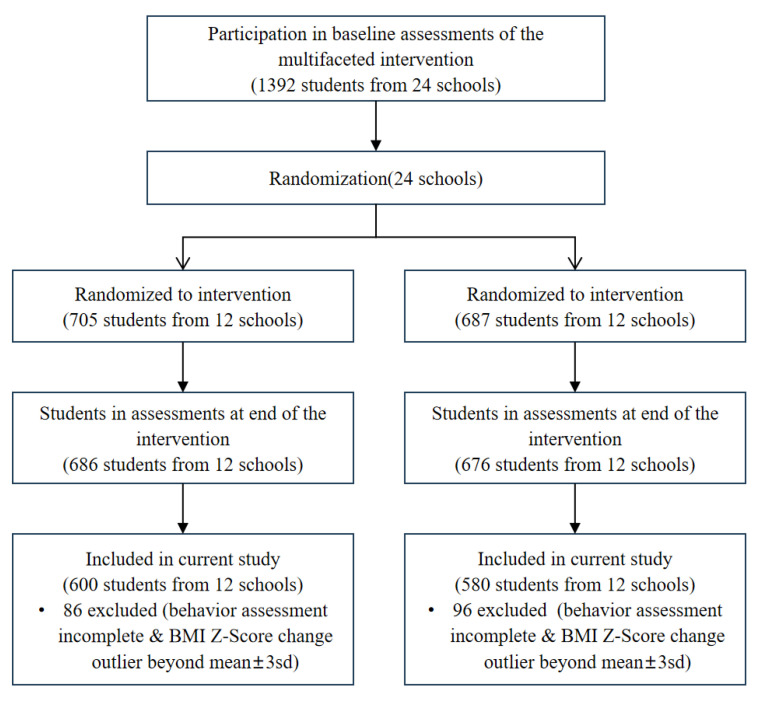
Flow diagram of selection of study participants.

Of the 1392 children who completed the baseline survey, 1362 eligible children finished the survey performed at the end of the trial [17]. In the present study, only those children who completed dietary behavior assessments at both baseline and the end of the trial were included. We also excluded children whose BMI z-score changes were outliers beyond mean ± 3 standard deviation (SD). Finally, a total of 1180 children who met the inclusion criteria were involved in this study (Figure 1).

### 2.2. Intervention

The DECIDE-Children study was developed to promote healthy eating and physical activity based on the social ecological model [29]. It was implemented over a whole school year between September 2018 and June 2019. The intervention focused on five components: Three of them targeted the children directly, including health education activities delivering 5 core messages (i.e., not eating excessively, not drinking sugar-sweetened beverages, less high-energy food, less sedentary time, and more physical activities), reinforcement of physical activity during class break and physical education lessons, and monitoring children’s height and weight through a smartphone application (Eat Wisely and Move Happily), which provided feedback about BMI status. The other two components targeted school and family environments, including instructing school principals/teachers and implementing school policies (e.g., not selling unhealthy snacks or sugar-sweetened beverages), delivering health education to parents, and teaching them to record their children’s dietary behavior and view autofeedback. The 12 control schools continued with their usual health education lessons and physical education sessions without additional interventions.

### 2.3. Measurements

#### 2.3.1. Assessment of Dietary Behaviors

Five dietary behaviors including consumption of sugar-sweetened beverages, fried food, Western fast food, unhealthy snacks, and eating out were considered. Consumption of sugar-sweetened beverages and unhealthy snacks was assessed by using a revised version of the Food Frequency Questionnaire (FFQ) [30,31], and their daily consumption was calculated by multiplying the daily intake frequency by the amount (milliliter or gram) consumed per occasion. Fried food, Western fast food, and eating out were measured by self-designed questions (How many days did you eat fried food/Western fast food/eat out during the last week?). All the information on these five dietary behaviors was collected both at baseline and at the end of the trial.

#### 2.3.2. Definition of Concurrent Changes in Dietary Behaviors

Each of the five dietary behaviors was categorized as eating or not eating both at baseline and at end of the trial, with not eating being defined as having achieved the goal of health education. One score was awarded for each behavior that achieved the goal, while behaviors that did not achieve the goal received a score of 0. The dietary behavioral score was defined by summing the score of each behavior at both baseline and the end of the trial. Based on the scores of each dietary behavior at baseline and at the end of the trial, each dietary behavior exhibited one of the four types of changes: consistently eating, beginning to eat, consistently not eating, and beginning not to eat. We described and analyzed all the adiposity indicator changes across different types of dietary behavior changes, and found that those with the same behavioral scores at the end of the trial had similar effect sizes of adiposity indicator changes (Table S1). Therefore, we combined consistently eating and beginning to eat into one category which was awarded for a score of 0, and consistently not eating and beginning not to eat into one category which was awarded for a score of 1 (Table S2). We adopted a composite score to investigate the degree of concurrent changes in multiple dietary behaviors. By summing the scores of each dietary behavior change, the total score of dietary behavioral change ranges from 0 to 5.

#### 2.3.3. Definition of Patterns in Dietary Behavioral Changes

Dietary behavioral change patterns were generated based on the scores of each dietary behavioral change, behaviors which were listed in the order of sugar-sweetened beverages, fried food, Western fast food, unhealthy snacks, and eating out, accordingly, with “0” representing consistently eating or beginning to eat, and “1” representing consistently not eating or beginning not to eat.

#### 2.3.4. Adiposity Outcomes

Adiposity outcomes in this study included BMI, BMI z-score, BF%, and WC. The children’s anthropometric measures at both baseline and the end of the trial through physical examination were conducted by trained personnel following a standard protocol. The methods about those indicators have been described in published articles [17]. BMI was calculated as weight in kilograms divided by height in square meters, and BMI z-score was calculated according to the World Health Organization (WHO) reference values [32]. Obesity was defined using age- and sex-specific BMI percentiles according to the Chinese reference values [33]. Changes in adiposity indicators were determined by subtracting the values at the end of the trial from the values at baseline.

### 2.4. Covariates

The children’s age and sex were collected at baseline by questionnaires. Considering that physical activity and screen time are associated with childhood obesity [34], changes in those behavior indicators were adjusted in the mediation analyses. The International Physical Activity Questionnaire Short Form (IPAQ-SF) was used to evaluate the children’s daily moderate-to-vigorous physical activity (MVPA) [35]. The self-designed question of “How much time on average did you spend watching television and playing on electronic devices every day during the last week?” was used to collect the children’s screen time. Children who engaged in moderate to vigorous physical activity for more than 1 h per day were considered to have met the goal. Screen time with less than 1 h per day was defined as achieving the goal.

### 2.5. Statistical Analyses

Continuous variables were described as means with SD, and categorical variables were presented as numbers and percentages (%). Basic characteristics were compared using the student’s *t* test for continuous data, with chi-square tests for categorical variables. One-way analysis of variance was used to examine differences in adiposity indicators across different types of dietary behavior changes, as well as differences of adiposity indicators across different dietary behavioral change patterns with the same scores of dietary behavioral changes.

To evaluate the effect of intervention on adiposity indicators and dietary behaviors, we used generalized linear mixed models which included school-level random intercept considering the correlation due to clustering of children within schools; adjusting for age, sex, and corresponding adiposity indicators; or dietary behavior at baseline. Generalized linear mixed models were also used to examine the association between scores of dietary behavioral changes and adiposity indicator changes, adjusting for children’s group assignment (intervention group or control group), age, sex, the corresponding behavior and adiposity indicators at baseline, changes in physical activity and screen time, and school clustering effect. Interaction terms between each dietary behavior variable and group assignment were used in the models to evaluate whether the effect of dietary behaviors on adiposity indicators varied significantly by group assignment. In addition, subgroup analyses were performed to examine whether the association between concurrent changes of dietary behaviors and changes in obesity indicators differed by sex and baseline BMI status. We also used interaction terms between each subgroup variable and scores of dietary behavioral changes to examine whether this effect varied by subgroups.

Mediation analysis was used to analyze the mediating effect of dietary behavioral change scores between intervention and adiposity indicator changes. Three regression analyses were conducted with adjustment of group assignment, age, sex, the corresponding dietary behavior and adiposity indicators at baseline, changes in physical activity and screen time, and a school-level random intercept being adjusted in all pathway modes in the mediation analysis [36]: Path A evaluated the intervention effect on the dietary behaviors at the end of the trial, Path B evaluated the association between dietary behaviors at the end of the trial and adiposity indicators with adjustment for the group assignment. Path C calculated the intervention effect on the adiposity indicators directly (total effect) and the coefficients of the intervention on the adiposity indicators when adjusting for the behaviors at the end of the trial (direct effect), while the product of coefficients in path A and B represented the indirect effect. The ratio of the indirect effect to the total effect was defined as the proportion of mediation.

The mediation effects of dietary behavioral score at the end of the trial on the associations between intervention and changes in adiposity indicators were estimated using the mediation package in R software. *p* < 0.05 was considered statistically significant. The data were analyzed using R software (version 4.4.1).

## 3. Results

### 3.1. Characteristics of Participants

The demographic characteristics of the included children were comparable to those of who were excluded (Table S3). The baseline characteristics were similar between the intervention group (*n* = 600) and the control group (*n* = 580) (Table 1) with no statistically significant differences observed (*p* > 0.05).

### 3.2. Effects of Dietary Behavioral Changes on Adiposity Indicators

We analyzed the intervention effect on adiposity indicators and dietary behaviors, and the results showed that intervention reduced all four adiposity indicators, and improved all five individual unhealthy dietary behaviors and dietary behavioral scores (Table S4).

**Figure 2:**
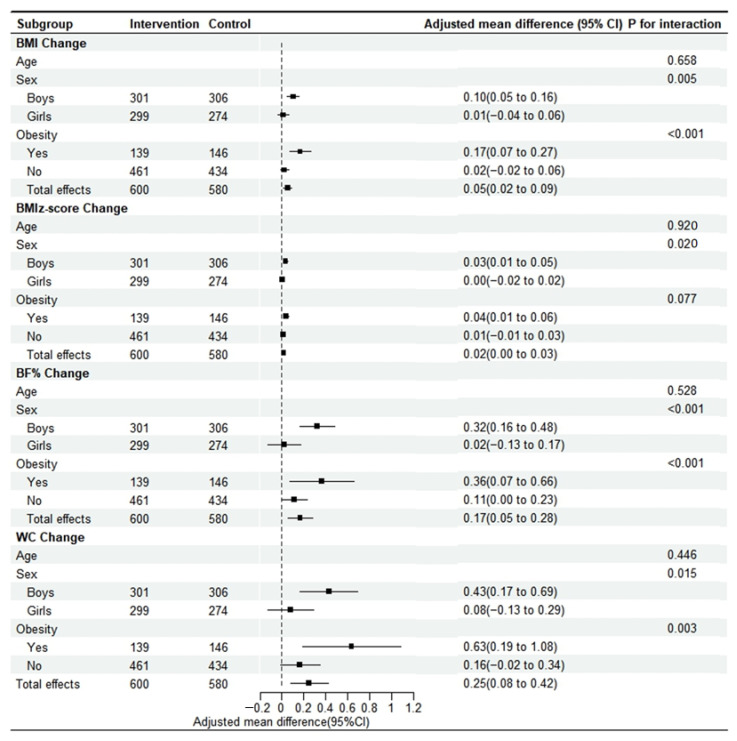
Subgroup analyses of the association between concurrent changes in dietary behaviors and changes in obesity indicators. Abbreviations: BF%, body fat percentage; BMI, body mass index; WC, waist circumference. Generalized linear mixed models were used, accounting for the school clustering effect, with adjustment for age, sex, corresponding behavior, and adiposity indicators at baseline. Changes in physical activity and screen time, interaction terms between each dietary behavior variable, and group assignment were used in the models.

Table 2 shows the association between scores of dietary behavioral changes and adiposity indicator changes. Results from individual behavioral analyses indicated that children who kept or began not to eat unhealthy snacks reduced more in BMI (0.21 [95% CI, 0.06 to 0.35]), BMI z-score (0.06 [95% CI, 0.01 to 0.12]) and BF% (0.49 [95% CI, 0.06 to 0.93]). Children who kept or began not to eat out also reduced more in BF% (0.50 [95% CI, 0.06 to 0.95]). Children who kept eating or began not to eat fried food (0.73 [95% CI, 0.06 to 1.40]) and not to eat out (0.88 [95% CI, 0.20 to 1.56]) decreased more in WC. Each increase in scores of dietary behavioral changes was associated with a 0.06 (95% CI, 0.01 to 0.11, *p* = 0.004) decrease in BMI change, 0.02 (95% CI, 0.001 to 0.04, *p* = 0.038) decrease in BMI z-score change, 0.21 (95% CI, 0.06 to 0.35, *p* = 0.005) decrease in BF% change, and 0.30 (95% CI, 0.08 to 0.53, *p* = 0.007) decrease in WC change. The results of the interaction analyses show that the effects of dietary behavioral changes on adiposity indicator changes did not differ significantly by group assignment (*p* > 0.05). As shown in Figure 2, the effect size did not differ significantly by age (*p* > 0.05 for interaction), but was stronger among boys than among girls, and among children who had obesity at baseline than among those who did not for all four adiposity indicators (*p* < 0.05 for interaction), except that the BMI z-score did not change significantly by baseline obesity (*p* > 0.05 for interaction).

### 3.3. Mediating Analyses

The single behavior mediation analyses results show that children who kept not eating or began not to eat unhealthy snacks have a significant mediated effect between the intervention and changes in BMI (7.26%, *p* = 0.002), BMI z-score (6.19%, *p* = 0.014), and BF% (7.80%, *p* = 0.008). Children who kept not eating or began not to eat fried food have a significant mediated effect between the intervention and changes in WC (9.34%, *p* =0.014) (Table 3).

The results considering overall changes in dietary behaviors indicated that scores of dietary behavioral changes mediated 13.87% (IE = 0.06, *p* = 0.002; DE = 0.34, *p* < 0.001), 11.81% (IE = 0.02, *p* = 0.028; DE = 0.12, *p* < 0.001), 17.60% (IE = 0.18, *p* < 0.001; DE = 0.79, *p* = 0.024), and 16.78% (IE = 0.26, *p* = 0.002; DE = 1.24, *p* = 0.032) of the association between the intervention and changes in BMI, BMI z-score, BF%, and WC, respectively (Table 3).

### 3.4. The Association Between Patterns of Dietary Behavioral Changes and Adiposity Indicator Changes

As shown in Table 4, there exist 24 patterns of dietary behavioral changes according to the scores of dietary behavioral changes. In total, 299 children (25.34%) kept or became free of five unhealthy dietary behaviors. There are four patterns with dietary behavioral changes scored 4 (23.22%), and a significant difference exists between those four patterns about BMI z-score change. Those children who kept not drinking or began not to drink sugar-sweetened beverages, not to eat fried food, not to eat Western fast food, and not to eat unhealthy snacks had the largest decrease in BMI z-score (0.27 ± 0.37). All four patterns have the common dietary behavior change type that children kept not eating or began not to eat Western fast food, and the pattern with children who kept not drinking or began not to drink sugar-sweetened beverages, not to eat Western fast food, not to eat unhealthy snacks, and not to eat out accounts for the largest proportion (32.8%). The number of patterns with dietary behavioral changes scored 1, 2, and 3 was 4, 7, and 7, respectively, and no significant differences were found among different patterns with different dietary behavioral change scores in all four adiposity indicators.

## 4. Discussion

In addition to the significant effectiveness of the high-quality, multifaceted DECIDE-Children trial on childhood obesity, our study further suggests that children with optimal dietary behavior after the intervention had greater reductions in BMI z-score, BMI, BF%, and WC. Mediation analyses also revealed that increased dietary behavioral change scores mediated the effect of intervention on changes in BMI z-score, BMI, BF%, and WC. To the best of our knowledge, this is the first study to explore how scores of dietary behavioral changes relate to adiposity indicators in obesity intervention studies among children, demonstrating the important role of concurrent changes in dietary behavior in the association between intervention and changes in adiposity indicators.

The analyses of individual dietary behaviors revealed that only changes in unhealthy snacking and fried food mediated the effect of intervention on adiposity indicator changes. Snacking behavior played the most pronounced mediating role in the children’s obesity among all dietary behaviors, with regard to the intervention effect on BMI z-score and BMI and BF% outcomes, while fried food behavior mainly mediated WC. On the one hand, this finding added evidence to the research gap in the association between unhealthy dietary behavioral changes and children’s adiposity outcome changes through data from RCT. On the other hand, although snacking and fried food behavior have been widely recognized as associated with childhood obesity [37,38], evidence from adult studies suggested that fried food was more closely associated with central obesity [39,40], which is defined by the World Health Organization as a WC of greater than 94 cm and 80 cm for males and females, respectively [41]. WC serves as a widely recognized and utilized indicator to assess the degree of fat accumulation in the abdomen [42]. Consequently, these findings that specific dietary behavior changes may be more closely associated with particular obesity outcomes will provide valuable evidence to support the development of dietary behavioral interventions for general and central obesity.

The dietary behavioral change score applied in the study showed that children with higher scores of dietary behavioral changes were significantly associated with greater reduction in adiposity indicators, and also indicated that scores of dietary behavioral changes mediated the effect of intervention on obesity outcomes. This finding not only reveals how interventions exert their effects but also informs future designs to attach importance to the joint effect of dietary behavior changes. Similar with conclusions from prevention gestational diabetes mellitus [43], this finding also contributes to the evidence that even a small change in dietary choices can be important when applied at a population level [44,45]. Considering the typical co-occurrence or clustering effect of multiple healthy or unhealthy behaviors [46,47], it is essential to address multiple unhealthy dietary practices in obesity intervention strategies.

The results of subgroup analyses revealed that the effect size differed significantly by sex and baseline BMI status, with the effect size of boys and children who had obesity at baseline larger than their counterparts. Evidence indicated that boys generally have higher basal metabolic rates and greater muscle mass compared to girls, which can lead to increased energy expenditure and more effective weight loss when dietary habits were improved at the same condition [48]. Children with obesity may have had more potential for improvement in their dietary behaviors, leading to more noticeable changes after the intervention. The severity of obesity might also have made them have more need for lifestyle changes, resulting in greater compliance and better outcomes [17,49].

Our dietary behavioral change pattern analyses revealed that there exist differences in the type and number of changes in dietary behavior among different children. This diversity suggests that each child may undergo a unique dietary behavior change trajectory, which may be influenced by a combination of biological, social, and environmental factors [50,51], except the intervention. This result further confirms that personalized intervention involving multiple dietary behavior goals should be developed to accommodate individual differences in children’s behavior changes. Furthermore, even in children with the same dietary behavioral change score of four, the significant difference in adiposity indicator changes among different dietary behavioral change patterns indicates that there may also be some optimal patterns. However, due to the limited sample size, this study could not draw a definitive conclusion in this regard. Future studies could further explore the optimal patterns of dietary behavior changes.

Family involvement [52] and school nutrition policies are effective components to shape children’s healthy dietary behavior [17,53,54]. Lifestyle interventions that engage both families and schools are essential for maximizing the number of unhealthy dietary behavioral changes in children, thereby leading to greater BMI and other adiposity indicators reduction. However, the current focus on children’s academic performance over physical fitness by families and schools in China has led to schools’ insufficient attention to childhood obesity prevention and insufficient cooperation from parents. Our successful APP-based, multifaceted DECIDE-Children trial provides an acceptable, timely, and effective mode to prevent childhood obesity in China and elsewhere in the future.

Our study has several strengths. First, it was conducted based on a well-designed and -executed multi-centered RCT that has been shown to be effective in improving children’s unhealthy dietary behavior and reducing obesity prevalence in primary school children. The RCT design is highly valued for its ability to minimize bias and confounding variables, providing robust evidence on the causal associations between concurrent changes of dietary behavior and changes in adiposity indicators [55]. Second, we conducted mediation analyses to identify whether scores of dietary behavioral changes acted as a significant mediator of the intervention effect. Our findings enhance the understanding of the mechanisms behind the beneficial effect of obesity intervention and inspire future research to strengthen the effective components of intervention. Third, we adjusted physical activity and screen time in the mediation analyses, which contributes to evaluating the mediating role of dietary behavior accurately. Last, we considered four adiposity indicators (BMI z-score, BMI, BF%, and WC) as outcomes, and the consistent findings further reinforce our conclusions.

This study also has some limitations. First, we were not able to conduct detailed comparisons across dietary behavioral change patterns due to the small sample sizes and insufficient statistical power in subgroups. However, the sample size was sufficient to address the main objectives of the present study. Second, while the children’s dietary behaviors were assessed using a revised FFQ and questionnaire with tailored questions capturing key health education elements, this method may not provide the precision of 24 h dietary recalls. Nonetheless, the FFQ has been widely used in large epidemiological studies since the 1990s. Its simplicity in assessing usual dietary intake, along with its cost-effectiveness and time-saving, makes it a valuable tool for epidemiological studies [56]. Third, although we adjusted for changes in physical activity and screen time in the analyses, they were not objectively measured in the present study, which may lead to recall bias and affect the reliability of the associations we observed. However, children and parents were guided by trained researchers, which reduced the bias to some extent.

## 5. Conclusions

A higher score of dietary behavioral changes was associated with more reductions in adiposity indicators in the children participating in the present RCT study. Optimal dietary behaviors targeted by the intervention significantly mediated its effectiveness in improving children’s adiposity indicators. Future childhood obesity intervention studies should promote multiple dietary behaviors simultaneously and further explore the most effective dietary behavioral change patterns for preventing childhood obesity.

## Figures and Tables

**Table 1:** Characteristics of participants at baseline.

Characteristic	Overall *N* = 1180	Intervention *n* = 600	Control *n* = 580	*p* ^1^
Region, *n* (%)				0.598
Beijing	413 (35.00)	216 (36.00)	197 (33.97)	
Changzhi, Shanxi Province	320 (27.12)	165 (27.50)	155 (26.72)	
Urumuqi, Xinjiang Province	447 (37.88)	219 (36.50)	228 (39.31)	
Sex, *n* (%)				0.373
Boys	607 (51.44)	301 (50.17)	306 (52.76)	
Girls	573 (48.56)	299 (49.83)	274 (47.24)	
Age (year), mean (SD)	9.63 (0.36)	9.62 (0.35)	9.64 (0.37)	0.592
BMI (kg/m^2^), mean (SD)	18.67 (3.68)	18.57 (3.73)	18.77 (3.63)	0.220
BMI z-score, mean (SD) ^2^	0.76 (1.42)	0.71 (1.44)	0.80 (1.40)	0.209
BF%, mean (SD)	20.86 (10.14)	20.69 (10.25)	21.03 (10.03)	0.445
WC (cm), mean (SD)	65.40 (10.57)	65.19 (10.30)	65.62 (10.84)	0.329
Obesity ^3^, *n* (%)				0.421
Yes	285 (24.15)	139 (23.17)	146 (25.17)	
No	895 (75.85)	461 (76.83)	434 (74.83)	

Abbreviations: BF%, body fat percentage; BMI, body mass index; SD, standard deviation; WC, waist circumference. ^1^ Differences between groups were tested by Chi-square tests for categorical variables, and Wilcoxon Rank Sum Test for continuous data. ^2^ BMI z-score was calculated based on World Health Organization (WHO) reference values. ^3^ Obesity was defined using age- and sex-specific BMI percentiles according to Chinese reference [33].

**Table 2:** The association between scores of dietary behavioral changes and adiposity indicator changes ^1^.

Scores of Dietary Behavioral Changes ^2^		BMI Change			BMI z-Score Change			BF% Change			WC Change		
		*β* (95% CI)	*p*	*p* Value for Interaction	*β* (95% CI)	*p*	*p* Value for Interaction	*β* (95% CI)	*p*	*p* Value for Interaction	*β* (95% CI)	*p*	*p* Value for Interaction
Sugar-sweetened beverages	0	ref			ref			ref			ref		
	1	0.04 (−0.10 to 0.19)	0.518	0.878	0.01 (−0.04 to 0.07)	0.590	0.914	0.30 (−0.13 to 0.74)	0.173	0.649	0.62 (−0.05 to 1.29)	0.069	0.536
Fried food	0	ref			ref			ref			ref		
	1	0.10 (−0.05 to 0.25)	0.181	0.666	0.03 (−0.02 to 0.09)	0.268	0.648	0.39 (−0.05 to 0.82)	0.085	0.367	0.73 (0.06 to 1.40)	0.033	0.744
Western fast food	0	ref			ref			ref			ref		
	1	0.09 (−0.08 to 0.26)	0.282	0.901	0.04 (−0.03 to 0.10)	0.259	0.790	0.31 (−0.21 to 0.82)	0.240	0.786	0.30 (−0.50 to 1.08)	0.470	0.443
Unhealthy snacks	0	ref			ref			ref			ref		
	1	0.21 (0.06 to 0.35)	0.005	0.387	0.06 (0.01 to 0.12)	0.024	0.509	0.49 (0.06 to 0.93)	0.028	0.717	0.26 (−0.41 to 0.94)	0.449	0.707
Eating out	0	ref			ref			ref			ref		
	1	0.10 (−0.05 to 0.25)	0.189	0.693	0.04 (−0.02 to 0.10)	0.169	0.345	0.50 (0.06 to 0.95)	0.027	0.173	0.88 (0.20 to 1.56)	0.011	0.084
Total scores of dietary behavioral changes ^3^		0.06 (0.01 to 0.11)	0.016	0.652	0.02 (0.001 to 0.04)	0.038	0.559	0.21 (0.06 to 0.35)	0.005	0.357	0.30 (0.08 to 0.53)	0.007	0.432

Abbreviations: Abbreviations: BF%, body fat percentage; BMI, body mass index; WC, waist circumference. ^1^ Generalized linear mixed models were used, accounting for the school clustering effect, with adjustment for age, sex, corresponding behavior and adiposity indicators at baseline, changes in physical activity and screen time; interaction terms between each dietary behavior variable and group assignment were used in the models. ^2^ Scores of dietary behavioral changes were defined with the type of consistently not eating or beginning not to eat scored 1, otherwise 0. ^3^ Total scores of dietary behavioral changes were calculated by summing the scores of each dietary behavior change, ranging from 0 to 5.

**Table 3:** Mediating role of scores of dietary behavioral changes in the intervention effects on changes in adiposity indicators ^1^.

Scores of Dietary Behavioral Changes ^2^	BMI Change		BMI z-Score Change		BF% Change		WC Change	
	Estimates (95% CI)	*p*	Estimates (95% CI)	*p*	Estimates (95% CI)	*p*	Estimates (95% CI)	*p*
Sugar-sweetened beverages								
Indrect effect	0.01 (−0.01 to 0.04)	0.340	0.00 (−0.01 to 0.02)	0.430	0.06 (−0.02 to 0.15)	0.162	0.12 (−0.01 to 0.27)	0.068
Direct effect	0.38 (0.16 to 0.60)	<0.001 ***	0.14 (0.05 to 0.22)	<0.001 ***	0.90 (0.20 to 1.61)	0.006 **	1.40 (0.26 to 2.55)	0.010 **
Total effect	0.39 (0.17 to 0.62)	<0.001 ***	0.14 (0.06 to 0.22)	<0.001 ***	0.96 (0.27 to 1.68)	0.002 **	1.52 (0.41 to 2.68)	0.004 **
Proportion of mediation, %	3.32 (−3.94 to 14.00)	0.340	2.85 (−5.38 to 15.00)	0.430	5.81 (−2.84 to 27.00)	0.164	7.46 (−0.68 to 34.00)	0.072
Fried food								
Indrect effect	0.02 (−0.01 to 0.04)	0.150	0.00 (0.00 to 0.01)	0.280	0.01 (−0.01 to 0.04)	0.340	0.14 (0.03 to 0.27)	0.006 **
Direct effect	0.37 (0.16 to 0.59)	<0.001 ***	0.13 (0.05 to 0.22)	<0.001 ***	0.38 (0.16 to 0.60)	<0.001 ***	1.35 (0.21 to 2.51)	0.018 *
Total effect	0.39 (0.18 to 0.61)	<0.001 ***	0.14 (0.06 to 0.22)	<0.001 ***	0.39 (0.17 to 0.62)	<0.001 ***	1.50 (0.39 to 2.65)	0.008 **
Proportion of mediation, %	4.13 (−1.60 to 15.00)	0.150	3.35 (−2.89 to 14.00)	0.280	3.32 (−3.94 to 14.00)	0.340	9.34 (2.07 to 41.00)	0.014 *
Western fast food								
Indrect effect	0.01 (0.00 to 0.04)	0.160	0.01 (0.00 to 0.01)	0.120	0.05 (−0.01 to 0.11)	0.100	0.06 (−0.02 to 0.16)	0.160
Direct effect	0.38 (0.16 to 0.60)	<0.001 ***	0.13 (0.05 to 0.22)	<0.001 ***	0.91 (0.21 to 1.63)	0.006 **	1.45 (0.29 to 2.62)	0.008 **
Total effect	0.39 (0.18 to 0.62)	<0.001 ***	0.14 (0.06 to 0.22)	<0.001 ***	0.96 (0.26 to 1.68)	0.002 **	1.51 (0.37 to 2.67)	0.006 **
Proportion of mediation, %	3.20 (−1.30 to 12.00)	0.160	3.85 (−1.03 to 14.00)	0.120	4.6 (−1.04 to 21.00)	0.102	3.88 (−1.64 to 19.00)	0.162
Unhealthy snacks								
Indrect effect	0.03 (0.01 to 0.06)	0.002 **	0.01 (0.00 to 0.02)	0.014 *	0.08 (0.01 to 0.17)	0.006 **	0.03 (−0.06 to 0.14)	0.498
Direct effect	0.36 (0.15 to 0.58)	<0.001 ***	0.13 (0.05 to 0.21)	<0.001 ***	0.89 (0.20 to 1.59)	0.006 **	1.48 (0.33 to 2.65)	0.006 **
Total effect	0.39 (0.18 to 0.61)	<0.001 ***	0.14 (0.06 to 0.22)	<0.001 ***	0.97 (0.28 to 1.67)	0.002 **	1.51 (0.39 to 2.67)	0.006 **
Proportion of mediation, %	7.26 (1.90 to 21.00)	0.002 **	6.19 (0.96 to 20.00)	0.014 *	7.80 (1.55 to 31.00)	0.008 **	1.86 (−5.10 to 15.00)	0.500
Eating out								
Indrect effect	0.02 (−0.01 to 0.05)	0.150	0.01 (−0.01 to 0.02)	0.320	0.08 (−0.01 to 0.18)	0.078	0.13 (−0.01 to 0.28)	0.068
Direct effect	0.37 (0.15 to 0.59)	<0.001 ***	0.13 (0.05 to 0.22)	<0.001 ***	0.88 (0.17 to 1.60)	0.010 **	1.38 (0.23 to 2.54)	0.014 *
Total effect	0.39 (0.18 to 0.62)	<0.001 ***	0.14 (0.06 to 0.22)	<0.001 ***	0.96 (0.27 to 1.68)	0.002 **	1.51 (0.38 to 2.68)	0.008 **
Proportion of mediation, %	5.26 (−2.09 to 19.00)	0.150	3.93 (−4.20 to 17.00)	0.320	7.86 (−0.99 to 34.00)	0.080	7.98 (−0.69 to 36.00)	0.076
Total scores of dietary behavioral changes ^3^								
Indrect effect	0.06 (0.02 to 0.10)	0.002 **	0.02 (0.00 to 0.03)	0.028 *	0.18 (0.05 to 0.31)	<0.001 ***	0.26 (0.07 to 0.47)	0.002 **
Direct effect	0.34 (0.12 to 0.55)	<0.001 ***	0.12 (0.04 to 0.21)	<0.001 ***	0.79 (0.08 to 1.49)	0.024 *	1.24 (0.10 to 2.40)	0.032 *
Total effect	0.39 (0.18 to 0.61)	<0.001 ***	0.14 (0.06 to 0.22)	<0.001 ***	0.96 (0.29 to 1.66)	0.004 **	1.51 (0.41 to 2.65)	0.006 **
Proportion of mediation, %	13.87 (3.74 to 36.00)	0.002 **	11.81 (1.51 to 34.00)	0.028 *	17.60 (4.93 to 66.00)	0.004 **	16.78 (4.45 to 69.00)	0.008 **

Abbreviations: BF%, body fat percentage; BMI, body mass index; WC, waist circumference. *** *p* < 0.001, ** *p* < 0.01, * *p* < 0.05. ^1^ Mediation analyses were used, accounting for the school clustering effect, with adjustment for group assignment, age, sex, corresponding behavior and adiposity indicators at baseline, changes in physical activity, and screen time. ^2^ Scores of dietary behavioral changes were defined with the type of consistently not eating or beginning not to eat scored 1, otherwise 0. ^3^ Total scores of dietary behavioral changes as a mediator were a continuous variable, which was calculated by summing the scores of each dietary behavior change, ranging from 0 to 5.

**Table 4:** Comparison of adiposity indicator changes between different patterns of dietary behavioral changes ^1^.

**Total Scores** **of Dietary Behavioral Changes**	**Patterns of Dietary** **Behavioral** **Changes ^2^**	**N (%)**	**BMI** **Change**	***p***	**BMI z-Score Change**	***p***	**BF%** **Change**	***p***	**WC** **Change**	***p***
0	0 0 0 0 0	78 (6.61)	−0.26 (0.82)		0.11 (0.34)		0.81 (2.88)		−0.49 (3.81)	
1	0 0 0 1 0	27 (2.29)	−0.33 (0.81)	0.843	0.06 (0.31)	0.611	0.39 (2.44)	0.532	−1.50 (2.92)	0.697
1	0 0 1 0 0	71 (6.02)	−0.21 (0.81)		0.14 (0.32)		0.82 (2.49)		−0.49 (6.43)	
1	0 1 0 0 0	5 (0.42)	−0.48 (0.50)		0.02 (0.20)		−0.76 (2.47)		−2.51 (1.96)	
1	1 0 0 0 0	29 (2.46)	−0.29 (0.93)		0.14 (0.36)		0.57 (2.56)		−1.33 (3.69)	
2	0 0 1 0 1	48 (4.07)	−0.18 (0.82)	0.918	0.17 (0.32)	0.889	0.82 (2.27)	0.859	−1.44 (4.17)	0.367
2	0 0 1 1 0	32 (2.71)	0.00 (0.92)		0.23 (0.34)		1.22 (2.90)		−3.03 (10.36)	
2	0 1 0 1 0	6 (0.51)	−0.19 (0.42)		0.12 (0.17)		0.65 (0.62)		−0.84 (1.65)	
2	0 1 1 0 0	30 (2.54)	−0.18 (0.81)		0.19 (0.32)		1.04 (2.53)		−0.29 (3.33)	
2	1 0 0 1 0	34 (2.88)	−0.08 (0.99)		0.16 (0.34)		1.31 (3.00)		−0.06 (3.92)	
2	1 0 1 0 0	30 (2.54)	−0.24 (0.79)		0.15 (0.29)		0.57 (2.92)		−1.01 (2.89)	
2	1 1 0 0 0	4 (0.34)	0.11 (0.71)		0.30 (0.36)		2.18 (1.48)		0.88 (4.82)	
3	0 0 1 1 1	32 (2.71)	0.14 (1.04)	0.685	0.28 (0.45)	0.866	1.01 (2.69)	0.562	0.24 (3.30)	0.733
3	0 1 1 0 1	30 (2.54)	−0.12 (0.76)		0.19 (0.27)		1.00 (2.07)		−0.18 (2.91)	
3	0 1 1 1 0	19 (1.61)	−0.18 (1.05)		0.15 (0.47)		0.77 (3.00)		−1.28 (2.95)	
3	1 0 1 0 1	49 (4.15)	−0.01 (0.80)		0.19 (0.31)		1.34 (2.30)		0.46 (3.21)	
3	1 0 1 1 0	53 (4.49)	−0.04 (0.87)		0.20 (0.36)		1.62 (2.92)		−0.15 (5.48)	
3	1 1 0 1 0	12 (1.02)	0.02 (0.36)		0.21 (0.21)		1.47 (0.96)		−0.56 (1.00)	
3	1 1 1 0 0	18 (1.53)	−0.31 (0.90)		0.15 (0.33)		0.31 (2.57)		0.56 (4.41)	
4	0 1 1 1 1	24 (2.03)	0.22 (1.10)	0.101	0.25 (0.32)	0.046	2.22 (3.26)	0.222	0.76 (2.97)	0.075
4	1 0 1 1 1	90 (7.63)	−0.07 (0.89)		0.19 (0.32)		1.30 (2.53)		0.01 (3.34)	
4	1 1 1 0 1	74 (6.27)	−0.20 (0.76)		0.12 (0.33)		1.01 (2.49)		−0.09 (3.00)	
4	1 1 1 1 0	86 (7.29)	0.12 (1.10)		0.27 (0.37)		1.69 (3.20)		1.06 (3.30)	
5	1 1 1 1 1	299 (25.34)	0.24 (0.88)		0.29 (0.34)		1.98 (2.91)		0.84 (3.68)	

Abbreviations: BF%, body fat percentage; BMI, body mass index; WC, waist circumference. ^1^ Differences were analyzed by one-way Analyses of Variance. ^2^ Dietary behaviors were listed in the order of sugar-sweetened beverages, fried food, Western fast food, unhealthy snacks, and eating out, accordingly, with “0” representing consistently eating or beginning to eat, with “1” representing consistently not eating or beginning not to eat.

## Data Availability

Data described in this manuscript will not be made available because the Peking University Institutional Review Board has not consented to this. Please contact the corresponding author at whjun@pku.edu.cn to access more information on data analyses.

## Supplementary Materials

The following supporting information can be downloaded at: https://www.mdpi.com/article/10.3390/nu17030376/s1, Table S1: Description and comparison of the four types of dietary behavior changes and adiposity indicators changes; Table S2: Description and comparison of the combined two types of dietary behavior changes and adiposity indicators changes; Table S3: Comparison of children who were included (*n* = 1180) and excluded (*n* = 212) in the present study; Table S4: Intervention effects on adiposity indicators and dietary behaviors achieved the goal at the end of the trial.
