# Supplementary material for: The Mediating Effect of Concurrent Changes in Dietary Behaviors on the Associations Between Intervention and Changes in Adiposity Outcomes: Evidence from a Cluster-Randomized Controlled Trial
Source: Nutrients. 2025 Jan 21;17(3):376. doi: 10.3390/nu17030376 (PMC11820463; doi:10.3390/nu17030376)
Supplement: Supplementary file 1 [file nutrients-17-00376-s001.zip › nutrients-3371242-supplementary.pdf]

Supplementary Materials

Table S1. Description and comparison of the four types of dietary behavior changes and adiposity indicators changes.

Table S2. Description and comparison of the combined two types of dietary behavior changes and adiposity indicators changes.

Table S3. Comparison of children who were included (*n*=1180) and excluded (*n*=212) in the present study.

Table S4. Intervention effects on adiposity indicators and dietary behaviors achieved the goal at the end of the trial

Table S1. Description and comparison of the four types of dietary behavior changes and adiposity indicators changes [1].

Type of dietary behavior changes	*n* (%)	BMI change	BMI z-score change	BF% change	WC change
Sugar-sweetened beverages					
Consistently drinking	200(16.95)	-0.17(0.82)	0.15(0.34)	0.77(2.51)	-0.90(4.29)
Beginning to drink	202(17.12)	-0.13(0.91)	0.17(0.34)	1.07(2.73)	-0.64(5.62)
Consistently not drinking	533(45.17)	0.01(0.89)	0.22(0.33)	1.44(2.78)	0.37(3.19)
Beginning not to drink	245(20.76)	0.12(0.93)	0.24(0.35)	1.73(2.88)	0.37(4.58)
p	—	0.002	0.016	0.001	<0.001
Fried food					
Consistently eating	353(29.92)	-0.11(0.90)	0.17(0.35)	1.05(2.69)	-0.24(4.18)
Beginning to eat	220(18.64)	-0.17(0.81)	0.16(0.31)	0.99(2.59)	-1.11(5.67)
Consistently not eating	320(27.12)	0.04(0.92)	0.23(0.35)	1.58(2.99)	0.47(3.49)
Beginning not to eat	287(24.32)	0.12(0.89)	0.25(0.34)	1.64(2.67)	0.54(3.43)
p	—	<0.001	0.002	0.004	<0.001
Western fast food					
Consistently eating	68(5.76)	-0.28(0.94)	0.09(0.35)	0.67(2.80)	-0.31(3.66)
Beginning to eat	127(10.76)	-0.19(0.76)	0.14(0.31)	0.91(2.56)	-0.94(3.44)
Consistently not eating	819(69.41)	0.01(0.89)	0.22(0.33)	1.42(2.78)	0.10(4.42)
Beginning not to eat	166(14.07)	0.05(0.94)	0.22(0.39)	1.44(2.78)	0.22(3.78)
p	—	0.006	0.002	0.047	0.056
Unhealthy snacks					
Consistently eating	261(22.12)	-0.20(0.76)	0.14(0.33)	0.90(2.45)	-0.51(3.58)
Beginning to eat	205(17.37)	-0.19(0.86)	0.16(0.31)	0.84(2.61)	-0.36(4.66)
Consistently not eating	430(36.44)	0.06(0.94)	0.23(0.34)	1.58(2.93)	0.11(4.68)
Beginning not to eat	284(24.07)	0.14(0.91)	0.26(0.37)	1.67(2.80)	0.48(3.53)
p	—	<0.001	<0.001	<0.001	0.023
Eating out					
Consistently eating	339(28.73)	-0.09(0.87)	0.19(0.34)	1.09(2.75)	-0.27(4.56)
Beginning to eat	195(16.53)	-0.20(0.94)	0.14(0.35)	0.97(2.87)	-0.77(5.36)
Consistently not eating	360(30.51)	0.04(0.85)	0.22(0.33)	1.48(2.78)	0.24(3.56)
Beginning not to eat	286(24.24)	0.11(0.92)	0.25(0.35)	1.65(2.65)	0.47(3.50)
p	—	<0.001	0.003	0.012	0.006
Total	1180(100)	-0.02(0.89)	0.21(0.34)	1.32(2.76)	-0.02(4.21)

Abbreviations: BF%, body fat percentage; BMI, body mass index; WC, waist circumference. [1] Differences between groups were tested by One-way analysis of variance.

Table S2. Description and comparison of the combined two types of dietary behavior changes and adiposity indicators changes [1].

Type of dietary behavior changes	*n* (%)	BMI change	BMI z-score change	BF% change	WC change
Sugar-sweetened beverages					
Not drinking at the end of the trial	402(34.07)	-0.15(0.87)	0.16(0.34)	0.92(2.63)	-0.77(5.00)
Drinking at the end of the trial	778(65.93)	0.04(0.90)	0.23(0.34)	1.53(2.81)	0.37(3.68)
p	—	<0.001	0.003	<0.001	<0.001
Fried food					
Not eating at the end of the trial	573(48.56)	-0.13(0.87)	0.17(0.34)	1.03(2.65)	-0.57(4.82)
Eating at the end of the trial	607(51.44)	0.08(0.91)	0.24(0.34)	1.61(2.84)	0.50(3.46)
p	—	<0.001	<0.001	<0.001	<0.001
Western fast food					
Not eating at the end of the trial	195(16.53)	-0.22(0.83)	0.12(0.32)	0.83(2.65)	-0.72(3.53)
Eating at the end of the trial	985(83.47)	0.02(0.90)	0.22(0.34)	1.42(2.78)	0.12(4.32)
p	—	<0.001	<0.001	0.006	0.011
Unhealthy snacks					
Not eating at the end of the trial	466(39.49)	-0.20(0.80)	0.15(0.32)	0.87(2.52)	-0.45(4.09)
Eating at the end of the trial	714(60.51)	0.09(0.93)	0.24(0.35)	1.62(2.88)	0.26(4.26)
p	—	<0.001	<0.001	<0.001	0.005
Eating out					
Not eating at the end of the trial	534(45.25)	-0.13(0.90)	0.17(0.34)	1.04(2.79)	-0.45(4.87)
Eating at the end of the trial	646(54.75)	0.07(0.88)	0.23(0.34)	1.56(2.72)	0.34(3.53)
p	—	<0.001	0.001	0.002	0.001
Total	1180(100)	-0.02(0.89)	0.21(0.34)	1.32(2.76)	-0.02(4.21)

Abbreviations: BF%, body fat percentage; BMI, body mass index; WC, waist circumference. [1] Differences between groups were tested by one-way analysis of variance.

Table S3. Comparison of children who were included (*n*=1180) and excluded (*n*=212) in the present study.

**Characteristics**	**Overall (n=1,392)**	**Included (n=1180）**	**Excluded (n=212）**	***p*** [2]
**Group, *n* (%)**				0.724
Intervention group	705(50.65)	600(50.85)	105(49.53)	
Control group	687(49.35)	580(49.15)	107(50.47)	
**Sex, *n* (%)**				0.905
Boys	717(51.51)	607(51.44)	110(51.89)	
Girls	675(48.49)	573(48.56)	102(48.11)	
Age (year), mean (SD)	9.63(0.36)	9.63(0.36)	9.6(0.36)	0.289
BMI (kg/m^2), mean (SD)	18.64(3.71)	18.67(3.68)	18.5(3.87)	0.569
BMI z-score, mean (SD)[1]	0.74(1.44)	0.76(1.42)	0.65(1.57)	0.344
BF%, mean (SD)	20.9(10.45)	20.86(10.14)	21.18(12.29)	0.700
WC (cm), mean (SD)	65.39(10.67)	65.4(10.57)	65.27(11.36)	0.879

Abbreviations: BF%, body fat percentage; BMI, body mass index; SD, standard deviation; WC, waist circumference. [1] BMI z-score was calculated based on World Health Organization (WHO) reference values. [2] Differences between groups were tested by chi-Square tests for categorical variables, and wilcoxon rank sum test for continuous data. Abbreviations: BMI, body mass index; BF%, body fat percentage; WC, waist circumference.

Table S4. Intervention effects on adiposity indicators and dietary behaviors achieved the goal at the end of the trial.

Outcomes	No.in intervention/ control group	Intervention group		Control group		Intervention vs control adjusted mean difference /OR (95% CI) [1]	*p*
		Baseline	End of trial	Baseline	End of trial		
**Adiposity outcomes**							
BMI (kg/m^2), mean (SD)	600/580	18.57 (3.73)	18.40 (3.68)	18.77 (3.63)	18.99 (3.79)	-0.40(-0.62 to -0.19)	0.001
BMI z-score, mean (SD)	600/580	0.71 (1.44)	0.43 (1.42)	0.80 (1.40)	0.67 (1.40)	-0.14(-0.22 to -0.06)	0.002
BF%, mean (SD)	600/580	20.70 (10.3)	18.90 (9.70)	21.00 (10.00)	20.20 (10.00)	-1.00(-1.71 to -0.29)	0.011
WC (cm), mean (SD)	600/580	65.19 (10.3)	64.54 (10.66)	65.62 (10.84)	66.33 (10.70)	-1.53(-2.67 to -0.39)	0.015
**Behavior outcomes at the end of the trial**							
Not drinking sugar-sweetened beverages, *n* (%)	600/580	364(60.67)	469(78.17)	371(63.97)	309(53.28)	3.65(2.33 to 5.73)	<0.001
Not eating fried food, *n* (%)	600/580	266(44.33)	374(62.33)	274(47.24)	233(40.17)	2.70(1.88 to 3.89)	<0.001
Not eating Western fast food, *n* (%)	600/580	483(80.50)	541(90.17)	463(79.83)	444(76.55)	2.86(1.96 to 4.18)	<0.001
Not eating unhealthy snacks, *n* (%)	600/580	303(50.50)	419(69.83)	332(57.24)	295(50.86)	2.60(1.57 to 4.30)	<0.001
Not eating out, *n* (%)	600/580	284(47.33)	412(68.67)	271(46.72)	234(40.34)	3.50(2.37 to 5.15)	<0.001
Dietary behavioral score, mean (SD)	600/580	2.83(1.44)	3.69(1.37)	2.95(1.42)	2.61(1.55)	1.11(0.78 to 1.43)	<0.001

Abbreviations: BF%, body fat percentage; BMI, body mass index; SD, standard deviation; WC, waist circumference. [1] Generalized linear mixed models were used, accounting for the school clustering effect, with adjustment for age, sex, and baseline values of the outcomes. Continuous data are calculated as mean between group difference; categorical variables are calculated as OR.
